# Critical role of TNF-α in cerebral aneurysm formation and progression to rupture

**DOI:** 10.1186/1742-2094-11-77

**Published:** 2014-04-16

**Authors:** Robert M Starke, Nohra Chalouhi, Pascal M Jabbour, Stavropoula I Tjoumakaris, L Fernando Gonzalez, Robert H Rosenwasser, Kosuke Wada, Kenji Shimada, David M Hasan, Nigel H Greig, Gary K Owens, Aaron S Dumont

**Affiliations:** 1 Joseph and Marie Field Cerebrovascular Research Laboratory, Division of Neurovascular and Endovascular Surgery, Department of Neurological Surgery, Thomas Jefferson University, Philadelphia, PA, USA; 2 Department of Neurological Surgery, University of Virginia, PO Box 800212, Charlottesville, VA 22908, USA; 3 Center for Cerebrovascular Research, University of California San Francisco, San Francisco, CA, USA; 4 Department of Neurosurgery, University of Iowa, Cedar Rapids, IA, USA; 5 National Institute on Aging, National Institutes of Health Translational Gerontology Branch, Intramural Research Program, District of Columbia, USA; 6 Department of Molecular Physiology and Biophysics, Robert M. Berne Cardiovascular Research Center, University of Virginia, Charlottesville, VA, USA; 7 Department of Neurological Surgery, Tulane University, New Orleans, LA, USA

**Keywords:** Cerebral, Aneurysm, Rupture, Subarachnoid hemorrhage, TNF-alpha, Tumor necrosis factor

## Abstract

**Background:**

Alterations in TNF-α expression have been associated with cerebral aneurysms, but a direct role in formation, progression, and rupture has not been established.

**Methods:**

Cerebral aneurysms were induced through hypertension and a single stereotactic injection of elastase into the basal cistern in mice. To test the role of TNF-α in aneurysm formation, aneurysms were induced in TNF-α knockout mice and mice pretreated with the synthesized TNF-α inhibitor 3,6′dithiothalidomide (DTH). To assess the role of TNF-α in aneurysm progression and rupture, DTH was started 6 days after aneurysm induction. TNF-α expression was assessed through real-time PCR and immunofluorescence staining.

**Results:**

TNF-α knockout mice and those pre-treated with DTH had significantly decreased incidence of aneurysm formation and rupture as compared to sham mice. As compared with sham mice, TNF-α protein and mRNA expression was not significantly different in TNF-α knockout mice or those pre-treated with DTH, but was elevated in unruptured and furthermore in ruptured aneurysms. Subarachnoid hemorrhage (SAH) occurred between 7 and 21 days following aneurysm induction. To ensure aneurysm formation preceded rupture, additional mice underwent induction and sacrifice after 7 days. Seventy-five percent had aneurysm formation without evidence of SAH. Initiation of DTH treatment 6 days after aneurysm induction did not alter the incidence of aneurysm formation, but resulted in aneurysmal stabilization and a significant decrease in rupture.

**Conclusions:**

These data suggest a critical role of TNF-α in the formation and rupture of aneurysms in a model of cerebral aneurysm formation. Inhibitors of TNF-α could be beneficial in preventing aneurysmal progression and rupture.

## Background

Cerebral aneurysm rupture is associated with significant morbidity and mortality [1,2]. A significant number of patients may be treated with microsurgery or endovascular coiling, but intervention is not without the risk of neurological injury [1,2]. A large number of patients are followed clinically as they are deemed either at lower risk of hemorrhage or at high risk for treatment. Even in these cohorts, a significant number of patients will go on to have aneurysmal rupture or receive treatment for aneurysm progression despite originally being considered high risk for intervention or low risk of rupture [2,3]. Medical therapy that stabilizes aneurysmal progression or rupture could be beneficial for a significant number of patients. Currently, there are no pharmacological alternatives for patients with cerebral aneurysms.

Inflammation has been implicated in the pathogenesis of intracranial aneurysm formation and rupture [4]. Alterations in TNF-α have been associated with cerebral aneurysm in humans [5,6], but a direct role in aneurysm formation or rupture has not been defined. TNF-α is a critical member of the immune system [7] and produces pro-inflammatory alterations in key cells implicated in cerebral aneurysms including macrophages, endothelial and smooth muscle cells [8-10].

The goals of the present study were to: 1) assess the direct role TNF-α in a model of cerebral aneurysm formation; 2) determine if there are alterations in TNF-α expression in cerebral aneurysm formation and rupture; 3) evaluate if TNF-α inhibition decreases the incidence of aneurysm formation; and 4) test whether TNF-α inhibition after cerebral aneurysm formation may lead to aneurysm stabilization and inhibition of rupture.

## Methods

This study was carried out in accordance with the recommendations in the *Guide for the Care and Use of Laboratory Animals* of the National Research Council [11]. The protocol was approved by the Institutional Animal Care and Use Committee of Thomas Jefferson University (Philadelphia, PA, USA). Cerebral aneurysms were induced in 8- to 10-week-old male TNF-α gene null (TNF-α −/−) mice (on C57BL/6 J background) or their wild type controls (Jackson Laboratory, Bar Harbor, ME, USA) using previously described methods [9,12,13] with alterations as herein described.

To induce hypertension, mice underwent nephrectomy followed by implantation of deoxycorticosterone acetate pellet (Innovative Research of America, Sarasota, FL, USA) 1 week later [14]. On the same day as deoxycorticosterone acetate pellet implantation, animals were started on water containing 1% NaCl (Sigma-Aldrich, St Louis, MO, USA) to induce hypertension [9,12-14] and 0.12% beta-aminoproprionitrile (BAPN; Sigma-Aldrich) to reduce collagen cross-linking [15]. Elastase (Sigma-Aldrich) was prepared in sterile PBS (Sigma-Aldrich). Mice underwent a single stereotactic elastase injection (35 mU) into the cerebrospinal fluid at the right basal cistern on the same day as pellet implantation [9,12,13]. Sham control mice received a single stereotactic injection of PBS. A single stereotactic injection of dye was performed for every 10 mice to ensure accurate needle placement. Animals were assigned to the sham or aneurysm induction cohorts randomly in an alternating fashion.

Blinded daily neurological examination was carried out using a previously described method [13,16-18]. Neurological symptoms were graded: 0, normal; 1, decreased drinking or eating with associated weight loss >2 g of body weight (approximately 10%) over 24 hours; 2, flexion of the torso and forelimbs on lifting of the animal by the tail; 3, circling to one side with a normal posture at rest; 4, leaning or falling to one side at rest; 5, no spontaneous activity. Mice were euthanized when they developed neurological symptoms (score 1 to 5). All asymptomatic mice were euthanized 28 days after aneurysm induction. The brain samples were perfused with PBS followed by a gelatin (Sigma-Aldrich) containing blue dye to visualize cerebral arteries as well as to assess for aneurysm formation and subarachnoid hemorrhage (SAH). Aneurysms were defined as a localized outward bulging of the vascular wall whose diameter was greater than 1.5 times the parent artery diameter by two independent observers blinded to the animal cohort [12,13]. Animal cohorts were not revealed until all experimental groups had been sacrificed.

Systolic blood pressure was measured by the tail-cuff method with the BP-2000 Blood Pressure Analysis System (Visitech Systems, Apex, NC, USA) after 3 days of training to allow for acclimation and then before aneurysm induction surgery (day 0) and every week until day 28 after surgery [19].

### Treatment with 3,6′dithiothalidomide (DTH)

To test whether TNF-α inhibition decreased the incidence of cerebral aneurysm formation, progression, and rupture, the TNF-α inhibitor 3,6′dithiothalidomide (DTH) was synthesized as previously described [20,21] and was greater than 99% purity. Sham animals and TNF-α knockout animals received intraperitoneal vehicle (1% carboxymethyl cellulose solution (Fluka, Sigma-Aldrich) prepared in sterile saline) and animals undergoing aneurysm induction surgery received intraperitoneal injections of the synthesized TNF-α inhibitor DTH [20,21], prepared as a suspension in the vehicle at a dose of 56 mg/kg. Dosing was based on preliminary studies and prior experiments [8,21]. To test whether TNF-α inhibition decreased the incidence of cerebral aneurysm formation, DTH treatment was started 3 days prior to elastase injection and continued for 28 days. To test whether TNF-α inhibition resulted in aneurysm stabilization and inhibition of rupture, DTH was started 6 days after elastase injection.

### Quantitative real-time PCR and immunohistochemistry

Quantitative real-time PCR and immunohistochemistry were carried out as previously described [8,22]. Details can be found in Additional file 1: Quantitative real-time PCR and immunohistochemistry.

### Statistical analysis

Primary outcomes were the incidence of aneurysm formation (both ruptured and unruptured) and the incidence of ruptured aneurysms. Further description of the methods of statistical analysis can be found in Additional file 1: Statistical analysis.

## Results

### Potential role of TNF-α in the formation of intracranial aneurysms

**Figure 1:**
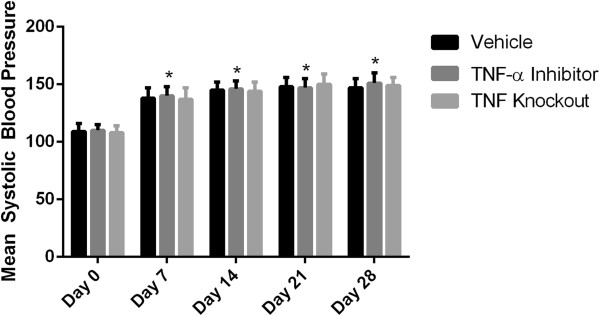
**Blood pressure analysis.** Blood pressure was elevated 1 week after aneurysm induction, but was not significantly different between mice pre-treated with DTH, TNF-α knockout mice, and mice receiving vehicle alone. DTH, 3,6'dithiothalidomide.

To test the role of TNF-α in the formation of intracranial aneurysms, the TNF-α inhibitor DTH was synthesized as previously described [20,21] to greater than 99% purity. Animals began treatment with DTH 3 days prior to elastase injection and were compared to both mice receiving vehicle undergoing aneurysm induction and TNF-α knockout mice undergoing aneurysm induction. TNF-α knockout mice, those treated with DTH, and those treated only with vehicle undergoing aneurysm induction had significant increases in systolic blood pressure 7 days after elastase injection that was sustained until 28 days, but was not significantly different between the three cohorts at any time point (Figure 1).

**Figure 2:**
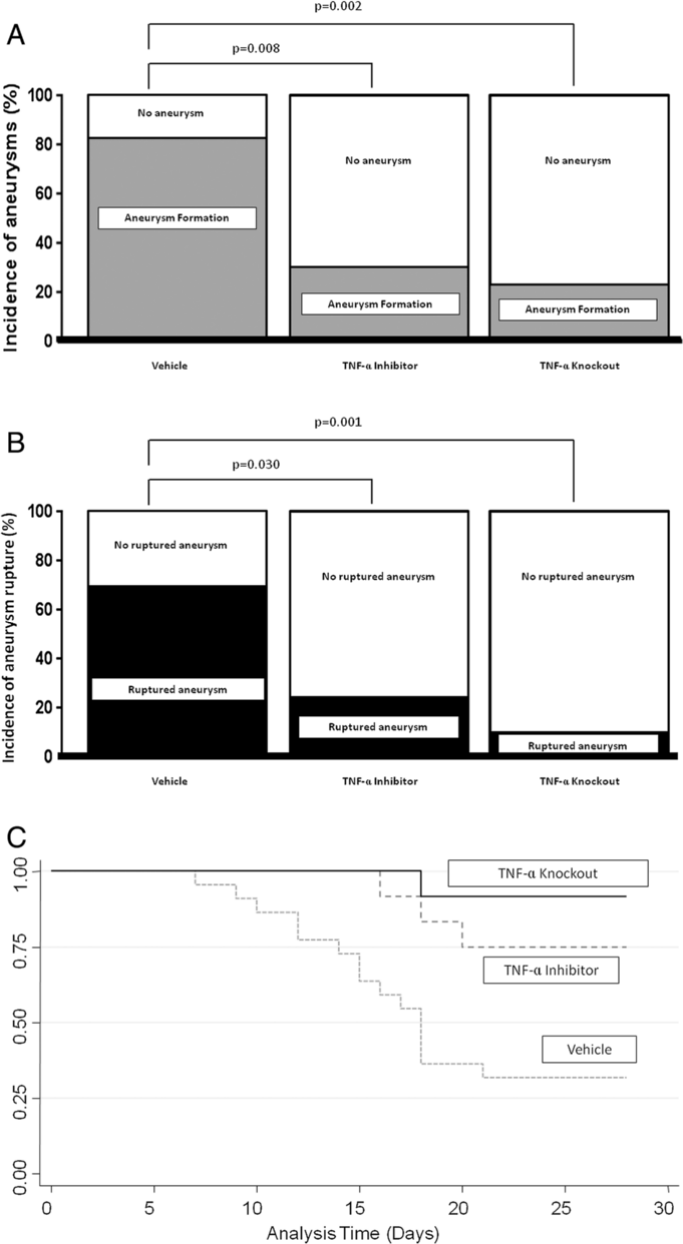
**The role of TNF-α in the formation of intracranial aneurysms. (A)** Cerebral aneurysm formation was significantly increased in mice receiving vehicle as compared to TNF-α knockout mice and those pre-treated with DTH. **(B)** The incidence of aneurysm rupture was also significantly increased in mice receiving vehicle versus TNF-α knockout mice and those pre-treated with DTH. **(C)** Kaplan-Meier analysis demonstrates that aneurysm rupture occurred between 7 and 21 days following aneurysm induction, and mice receiving vehicle were more likely to have ruptured aneurysms as compared to TNF-α knockout mice and those pre-treated with DTH. DTH, 3,6'dithiothalidomide.

Cerebral aneurysm formation occurred in 18 of 22 (81.8%) animals receiving only vehicle as compared to 3 of 12 TNF-α knockout mice (25%, *P* = 0.002) and 4 of 12 mice (33%, *P* = 0.008) treated with DTH (Figure 2A). The incidence of ruptured aneurysm was also significantly increased in those receiving vehicle (15 of 22, 68.2%) as compared with TNF-α knockout mice (1 of 12, 8.3%; *P* = 0.001) and those receiving DTH (3 of 12, 25%, *P* = 0.030; Figure 2B). Animals developed new neurological signs (score 1 to 5) and aneurysmal SAH between 7 and 21 days (Figure 2C). TNF-α knockout mice were 12.4 times (95% CI 1.6 to 94.2, *P* = 0.015) and those treated with DTH were 4.1 times (95% CI 1.2 to 14.1, *P* = 0.028) less likely to have aneurysm rupture as compared to mice receiving vehicle.

### TNF-α expression in unruptured and ruptured intracranial aneurysms

**Figure 3:**
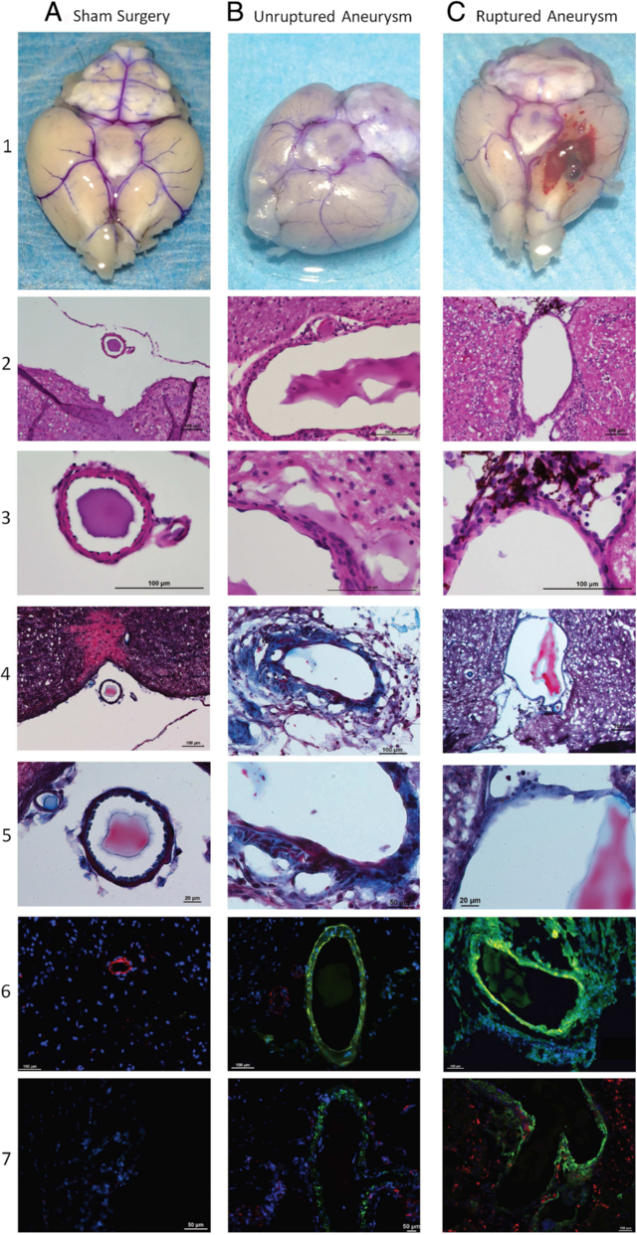
**Cerebral aneurysm formation and rupture and TNF-α expression.** Representative gross images of normal cerebral blood vessels in **(A)** sham operated mice, **(B)** unruptured aneurysms, and **(C)** ruptured aneurysms. H & E staining of normal cerebral blood vessels in sham operated mice demonstrates two to three layers of smooth muscle cells and a single, thin, continuous layer of endothelial cells (A2 to 3). In both unruptured cerebral aneurysms (B2 to 3) and ruptured aneurysms (C2 to 3) there are thin and thick areas of vascular wall. In unruptured aneurysms in the areas of thin vascular wall, there is intact endothelial and smooth muscle cell layers, but this is discontinuous in the areas of thick vascular wall. In ruptured aneurysms these areas are sometimes discontinuous in both thick and thin areas of the vascular walls. Trichrome staining of normal cerebral blood vessels in sham operated mice (A4 to 5) demonstrates one layer of elastic lamina. This is disorganized in unruptured aneurysms (B4 to 5) and ruptured aneurysms (C2 to 3). TNF-α (green) expression is nearly non-existent in sham operated mice (A6 to 7). The expression of TNF-α (green) is decreased in sham mice (A6 to 7) as compared to unruptured (B6 to 7) and furthermore ruptured aneurysms (C6 to 7). SMC-22α (red) is observed in sham operated mice (A6) and co-localizes with TNF-α (green) to appear yellow in unruptured (B6) and ruptured aneurysms (B7). Normal sized cerebral blood vessels also stain only red for SM-22α in vascular segments adjacent to cerebral aneurysms (B6). Macrophages are nearly non-existent in sham operated mice (A7). Macrophages (red) co-localize with TNF-α (green) and appear yellow in unruptured aneurysms (B7) and more so in ruptured aneurysms (C7). Nuclei are stained blue with DAPI. DAPI, 4',6-diamidino-2-phenylindole; H & E, hematoxylin and eosin; SM-22α, smooth muscle cell 22 alpha.

Figure 3A shows the normal brain vasculature from a mouse following sham surgery. This was not significantly different from mice pre-treated with DTH or TNF-α knockout mice (Additional file 1: Figure S1). An unruptured intracranial aneurysm from a mouse sacrificed on day 28 is depicted in Figure 3B. Figure 3C demonstrates a ruptured intracranial aneurysm that was found on post-operative day 12 following aneurysm induction in a mouse that presented with acute onset of crouching and hemiparesis manifesting as circling to the right. Most of the aneurysms were larger than 500 μm, approximately 3 to 5 times larger than their parent arteries.

Histological assessment of the aneurysms demonstrated structural changes that were similar to those found in human cerebral aneurysms (Figure 3) [12,23]. H & E and trichrome staining of cerebral vessels from sham operated mice demonstrated two to three layers of smooth muscle cells and a single layer of continuous endothelial cells (A2 to 5). Trichrome staining demonstrated one layer of elastic lamina as previously noted (A4 to 5) [12]. In intracranial aneurysms, there were layers of discontinuous endothelial cells and scattered smooth muscle cells (B2 to 5 and C2 to 5). Trichrome staining revealed disorganized elastic lamina (B4 to 5 and C4 to 5).

As alterations in TNF-α have been associated with human intracranial aneurysms [5,6], we sought to assess alterations in expression of TNF-α in this animal model of cerebral aneurysms. As compared to sham mice, there was no difference in immunofluorescence reactivity when comparing TNF-α knockout mice or those treated with DTH (Additional file 1: Figure S1). TNF-α expression was higher in unruptured aneurysms and highest in ruptured aneurysms (Figure 3), but this may be due to inflammation caused by SAH rather than increased TNF-α expression leading to aneurysmal rupture. As we have previously found that smooth muscle cells can produce TNF-α [8], we sought to determine the source of TNF-α. To localize TNF-α expression, samples were co-stained with either SMC-22α for smooth muscle cells or CD-68 for macrophages (Figure 3). TNF-α expression co-localized to both smooth muscle cells and macrophages in both unruptured (B6 to 7) and ruptured aneurysms (C6 to 7). Normal sized cerebral blood vessels also stain only red for SM-22α in vascular segments adjacent to cerebral aneurysms (B6). There were more macrophages in ruptured aneurysms (C7) as compared to unruptured aneurysms (B7) and an absence of macrophages in sham operated mice (A7).

**Figure 4:**
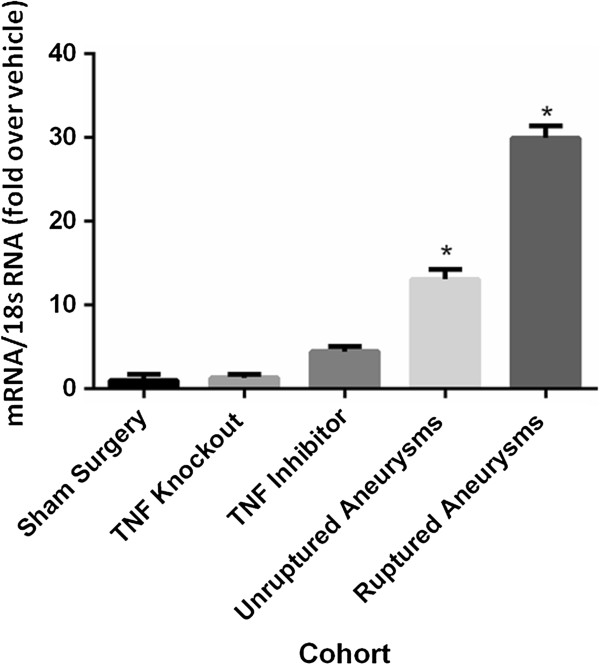
**TNF-α mRNA expression.** As compared with sham mice receiving vehicle, real-time PCR demonstrated no significant difference in TNF-α mRNA expression of cerebral blood vessels from TNF-α knockout mice or those pre-treated with DTH, but TNF-α was significantly elevated in unruptured and furthermore in ruptured aneurysms. DTH, 3,6'dithiothalidomide.

Similarly, as compared with sham mice, real-time PCR demonstrated no significant difference in TNF-α mRNA expression of cerebral blood vessels from TNF-α knockout mice or those treated with DTH (Figure 4). TNF-α mRNA extracted from unruptured, and even more so in ruptured aneurysms, was significantly elevated as compared with sham mice (Figure 4). As a secondary control, beta-actin expression was assessed and found to be not significantly different between sham mice, TNF-α knockout mice, mice treated with DTH, unruptured aneurysm, and ruptured aneurysms (Additional file 1: Figure S2).

### Timing of cerebral aneurysm formation

Prior studies have found that cerebral aneurysm formation in a similar model of aneurysm formation occurs between 7 and 21 days [13]. To further define the time course of aneurysm formation and verify that aneurysmal formation occurs prior to aneurysmal rupture, eight additional mice underwent aneurysm induction. None of the mice developed neurological alterations during this time period. Mice were euthanized 7 days after elastase injection at a time point before aneurysm rupture in the prior cohort of animals. Six of eight animals (75%) formed aneurysms and none had evidence of aneurysmal rupture. The overall incidence of aneurysm formation in this cohort was similar to the rate of aneurysms in the prior untreated cohort (81.8%). Additionally, this confirms that from day 7 to day 21 appears to represent a time-frame during which medical therapies targeting aneurysm stabilization can be tested without altering the formation of aneurysms.

### Effect of TNF-α inhibition on the incidence of aneurysm rupture

**Figure 5:**
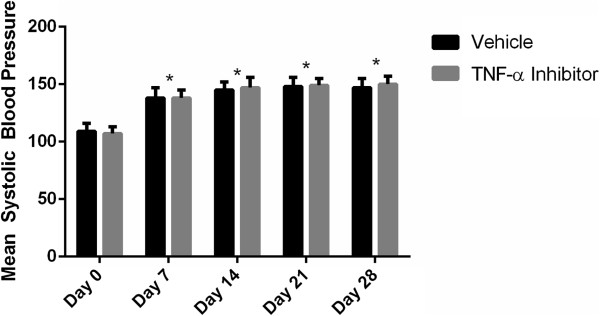
**Blood pressure analysis in delayed DTH treatment.** Blood pressure was elevated 1 week after aneurysm induction, but was not significantly different between mice treated with DTH starting on day 7 and mice receiving vehicle alone. DTH, 3,6'dithiothalidomide.

To test whether TNF-α inhibition could stabilize aneurysm progression and decrease the incidence of aneurysm rupture, DTH was started 6 days after elastase injection. Those treated with DTH and mice receiving vehicle undergoing aneurysm induction had significant increases in systolic blood pressure 7 days after elastase injection that was sustained until 28 days, but was not significantly different between the two cohorts at any time point (Figure 5).

**Figure 6:**
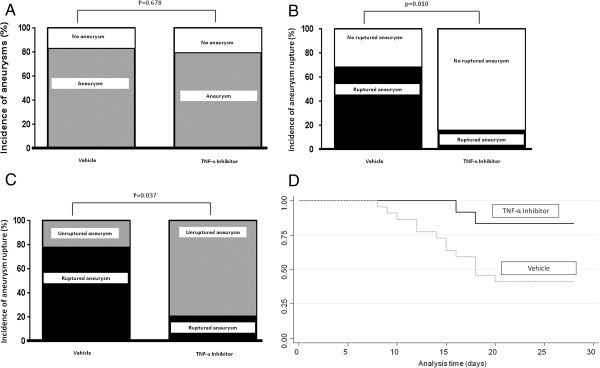
**Aneurysm stabilization with DTH. (A)** There was no significant difference in the overall incidence of aneurysm formation between cohorts treated with DTH and those treated with vehicle. **(B)** The incidence of rupture was significantly decreased in those treated with DTH as compared to vehicle. **(C)** The rate of cerebral aneurysm rupture in those with cerebral aneurysm formation was significantly decreased in those treated with DTH as compared with vehicle. **(D)** After eliminating those animals without aneurysm formation, Kaplan-Meier analysis demonstrated that those receiving delayed DTH treatment were 5.8 times (95% CI 1.3 to 25.5, *P* = 0.020) less likely to have aneurysmal rupture than those receiving vehicle. DTH, 3,6'dithiothalidomide.

There was no significant difference in the overall incidence of aneurysm formation between cohorts treated with DTH (9 of 12, 75%) and those treated with vehicle (18 of 22, 83%, *P* = 0.678; Figure 6A). The incidence of rupture was significantly decreased in those treated with DTH (2 of 12, 16.7%) as compared to vehicle (15 of 22, 68.2%, *P* = 0.010; Figure 6B). Additionally, the rate of cerebral aneurysm rupture in those with cerebral aneurysm formation was significantly decreased in those treated with DTH (2 of 9, 22.2%) as compared with vehicle (15 of 18, 83.3%, *P* = 0.037; Figure 6C). A symptom-free Kaplan-Meier analysis was carried out to determine the effect of DTH on the overall incidence of rupture. After eliminating those animals without aneurysmal formation, those receiving delayed DTH treatment were 5.8 times less likely to have aneurysmal rupture than those receiving vehicle (95% CI 1.3 to 25.5, *P* = 0.020; Figure 6D).

## Discussion

Cerebral aneurysm rupture leads to disability or death in the majority of patients [1,2]. Treatment is also associated with significant morbidity and mortality, particularly in high risk aneurysms or patients [1,2]. Although intervention is controversial in select patients with unruptured aneurysms, studies have found that a large number of patients deemed low risk of hemorrhage or high risk for treatment may go on to receive treatment for aneurysm progression or experience SAH [2,3]. Currently, there are no medical therapies in clinical practice to stabilize aneurysmal progression or prevent rupture. In this study we have found that the expression of the pro-inflammatory cytokine TNF-α is increased in a mouse model of cerebral aneurysms. This expression is increased in unruptured cerebral aneurysms and furthermore in ruptured aneurysms. The incidence of cerebral aneurysm formation and rupture was decreased in TNF-α knockout mice and following pre-treatment with a synthesized TNF-α inhibitor. The TNF-α inhibitor also resulted in aneurysm stabilization and decreased rupture after aneurysm formation.

TNF-α has also been found to be elevated in humans with ruptured cerebral aneurysms [5,6], but mechanisms behind TNF-α activation in cerebral aneurysms are currently unclear. A number of environmental factors associated with cerebral aneurysm formation have been implicated in TNF-α activation. We have previously found that induced hypertension in conjunction with hemodynamic stress in rats *in vivo* can increase the expression of TNF-α [8]. We have also found that TNF-α is upregulated following exposure of cultured cerebrovascular smooth muscle cells to cigarette smoke [22,24], and others have found increased expression of TNF-α in blood vessels following cigarette exposure [25]. Additional risk factors associated with cerebral aneurysms, including aging, gender, and alcohol, have also been associated with TNF-α expression [4-6].

Environmental factors may activate TNF-α in both macrophages and smooth muscle cells. In this study we have found that TNF-α co-localized to both macrophages and smooth muscle cells. We have previously found that hypertension, hemodynamic stress, and cigarette smoke may activate TNF-α and induce phenotypic modulation in smooth muscle cells [8]. Although the downstream mechanisms by which TNF-α contributes to cerebral aneurysm formation are not completely defined, inflammation is a significant element behind the pathogenesis of cerebral aneurysm formation [4], and TNF-α is a significant pro-inflammatory immune modulator. We have previously found that TNF-α can activate a number of pro-inflammatory and matrix remodeling genes in cerebral vascular smooth muscle cells directly implicated in cerebral aneurysm formation, including: MMP-3, MMP-9, MCP-1, VCAM-1, and IL-1β [8]; and IL-1β, VCAM-1 and MCP-1 [9,26,27]. Specifically, increased MCP-1 may attract macrophages leading to increased TNF-α formation, and macrophages have previously been found to be critical in the formation of cerebral aneurysms [9]. Additionally, MCP-1 has been found to be increased in aneurysm walls and MCP-1 knockout mice had lower levels of matrix metalloproteinases (MMPs), and a decreased incidence of aneurysm formation [26]. MMPs degrade the extracellular matrix [28] and have been found to be increased in human cerebral aneurysms [29]. Inhibition of MMPs has also been shown to decrease the incidence of aneurysm formation and progression in animals [12,13]. Although there are likely many further key mediators, TNF-α may contribute to cerebral aneurysm formation through activation of pro-inflammatory and matrix remodeling genes and recruitment and activation of inflammatory cells.

The role of TNF-α in aneurysm progression and rupture is unknown. Increased expression of TNF-α in human [5,6] and mouse ruptured cerebral aneurysms may be a reflection of the inflammatory response following rupture rather than a mechanism leading to aneurysm instability and rupture. TNF-α is a key initiator of apoptosis [30,31] and its pro-apoptotic protein target (Fas-associated death domain) have been increased in human cerebral aneurysms [5]. We have previously found that TNF-α triggers apoptosis in a dose-dependent fashion in cultured cerebrovascular smooth muscle cells [8], and this may contribute to both aneurysm progression and rupture through loss of focal cerebrovascular contractility. This may be explained by the co-localization of TNF-α to smooth muscle cells in cerebral aneurysms found in mice and ultimately the loss of smooth muscle cells in unruptured and furthermore ruptured aneurysms. TNF-α-induced macrophage infiltration and phagocytosis may also contribute to this process [26,32] as the quantity of apoptotic cells has been associated with cerebral aneurysm rupture [33,34] and macrophage infiltration was increased furthermore in ruptured versus unruptured aneurysms.

Additional experiments are indicated both *in vivo* and in humans to assess the role of TNF-α in cerebral aneurysm progression and rupture. Further *in vivo* experiments following upregulation of TNF-α both in wild type and TNF-α knockout mice would be beneficial to further clarify the molecular mechanisms downstream of TNF-α upregulation in aneurysm formation and rupture. Limitations of this study include detection of aneurysm rupture through assessment of alterations in neurological examination, which may fail to detect subclinical or asymptomatic hemorrhages. Despite this drawback, examination of brains in asymptomatic mice did not reveal signs of significant SAH. Additionally, although DTH has been found to be a specific inhibitor of TNF-α synthesis [20,21], it may have additional properties that contribute to inhibition of aneurysm formation and rupture.

## Conclusions

In summary, we have demonstrated that TNF-α is increased in both unruptured and ruptured cerebral aneurysms in an *in vivo* model of cerebral aneurysm formation. TNF-α expression was decreased in TNF-α knockout mice, those pre-treated with the synthesized TNF-α inhibitor DTH, and controls versus unruptured and ruptured aneurysms. Additionally, the incidence of aneurysm formation and rupture was significantly decreased in TNF-α knockout mice and those pre-treated with DTH. Finally, treatment with DTH after aneurysm formation resulted in aneurysm stabilization and markedly decreased the incidence of rupture.

## Abbreviations

BAPN: Beta-aminoproprionitrile; DAPI: 4′,6-Diamidino-2-phenylindole; DTH: 3,6′Dithiothalidomide; H & E: Hematoxylin and eosin; IL-1β: Interleukin 1-beta; MCP-1: Monocyte chemoattractant protein-1; MMP: Matrix metalloproteinase; PBS: Phosphate buffered saline; PCR: Polymerase chain reaction; SAH: Subarachnoid hemorrhage; SM-22α: Smooth muscle cell 22 alpha; TNF-α: Tumor necrosis factor alpha; VCAM-1: Vascular cell adhesion molecule-1.

## Competing interests

The authors declare that they have no competing interests.

## Authors’ contributions

RS and AD made substantial contributions to conception and design, carrying out experiments, acquisition of data, and analysis and interpretation of data. NC, KW, and KS made substantial contributions to carrying out experiments, acquisition of data, and analysis and interpretation of data. PJ, ST, LFG, RR, DH, and GO made substantial contributions to conception and design, and analysis and interpretation of data. NG made substantial contributions to conception and design, and carrying out experiments. All authors were involved in drafting the manuscript and revising it critically for important intellectual content; have given final approval of the version to be published; and agree to be accountable for all aspects of the work in ensuring that questions related to the accuracy or integrity of any part of the work are appropriately investigated and resolved.

## Supplementary Material

Additional file 1 **Quantitative real-time PCR, immunohistochemistry, and statistical analysis. Figure S1.** TNF-α expression in TNF-α knockout mice and those pretreated with DTH. **Figure S2.** Beta-actin mRNA expression. Click here for file
