# Supplementary material for: Critical role of TNF-α in cerebral aneurysm formation and progression to rupture
Source: J Neuroinflammation. 2014 Apr 16;11:77. doi: 10.1186/1742-2094-11-77 (PMC4022343; doi:10.1186/1742-2094-11-77)
Supplement: Additional file 1 — Quantitative real-time PCR, immunohistochemistry, and statistical analysis. Figure S1. TNF-α expression in TNF-α knockout mice and those pretreated with DTH. Figure S2. Beta-actin mRNA expression. [file 1742-2094-11-77-S1.doc]

**SUPPLEMENTAL ONLINE METHODS and RESULTS**

**Critical Role of TNF-α in Cerebral Aneurysm Formation and**

**Progression to Rupture**

Robert M. Starke, M.D., M.Sc.,^1,2^ Nohra Chalouhi, M.D.,^1^ Pascal M. Jabbour, M.D.,^1^ Stavropoula I. Tjoumakaris, M.D.,^1^ L. Fernando Gonzalez, M.D.,^1^ Robert H. Rosenwasser, M.D.,^1^ Kosuke Wada, M.D.,^3^ Kenji Shimada, M.D.^3^ David M. Hasan, M.D.,^4^ Nigel H. Greig Ph.D.,^5^ Gary K. Owens, Ph.D.,^6^ Aaron S. Dumont, M.D.^1,7^

*From the ^1^Joseph and Marie Field Cerebrovascular Research Laboratory, Division of Neurovascular & Endovascular Surgery, Department of Neurological Surgery, Thomas Jefferson University ^2^Department of Neurological Surgery, University of Virginia,^3^ Center for Cerebrovascular Research, University of California San Francisco, ^4^Department of Neurosurgery, University of Iowa, ^5^National Institute on Aging, National Institutes of Health Translational Gerontology Branch, Intramural Research Program ^6^Department of Molecular Physiology and Biophysics, Robert M. Berne Cardiovascular Research Center, University of Virginia, ^7^Department of Neurological Surgery, Tulane University*

**Quantitative Real-Time RT-PCR**

Cerebral aneurysms or cerebral blood vessels in sham mice without cerebral aneurysm formation were extracted and homogenized using Ultra-Turrax (Sigma-Aldrich), and total RNA was extracted using RNeasy fibrous mini kit (Qiagen) using manufacturer’s protocol. cDNA was prepared using 0.5-1 μg of total RNA using iScript cDNA synthesis kit (Bio-Rad laboratories). iQ SYBR Green (Bio-Rad laboratories) was used to run quantitative real-time polymerase chain reaction (RT-PCR) using a CFX-96 real time system (Bio-Rad laboratories). The following forward and reverse TNF-α primer sequences were used, respectively, *AAAGCATGATCCGAGATGT* and *AGCAGGAATGAGAAGAGGC*. Results were normalized to 18S RNA gene expression.

**Immunohistochemistry**

Animal brains were placed in 4% paraformaldehyde for 24 hours and then immersed in 15% sucrose for 24 hours and 30% sucrose for another 24 hours. The tissue was then frozen in OCT compound (Tissue-Tek) at -80°C. Serial cross-sections (10 μm thick) were mounted on microscope slides (Fisher Scientific Co.). After blocking for 2 hours with 5% donkey serum, 10 μm frozen sections were incubated with primary antibodies (anti-TNFα, ABCAM; anti-SM-22α, ABCAM; anti-CD68, Serotec) overnight followed by fluorescent-labeled secondary antibodies (Alexa Fluor; Life Technologies) for 2 hours at room temperature. Nuclei were counter-stained with DAPI (Sigma Aldrich). The slides were then observed under a fluorescent microscope (Carl Zeiss, Axio Imager). Slides were assessed by two independent observers blinded to animal cohort.

**Statistical Analysis**

Primary outcomes were the incidence of aneurysm formation (both ruptured and unruptured) and the incidence of ruptured aneurysms (number of mice with ruptured aneurysms/number of mice with ruptured or unruptured aneurysms). Test of proportions was carried out using Fisher exact test for the primary analysis. Secondary assessment of risk of rupture was carried out by Kaplan-Meier survival analysis. The log-rank test was used to assess differences in survival curves and Cox regression was used to assess hazard ratios. Mean systolic blood pressure between two groups was compared using Wilcoxon rank-sum test and Kruskal-Wallis one-way analysis of variance in three groups. Statistical significance was considered at P<0.05.

**Additional file1: Figure S1** **TNF-α expression in TNF-α knockout mice and those pretreated with DTH**

**
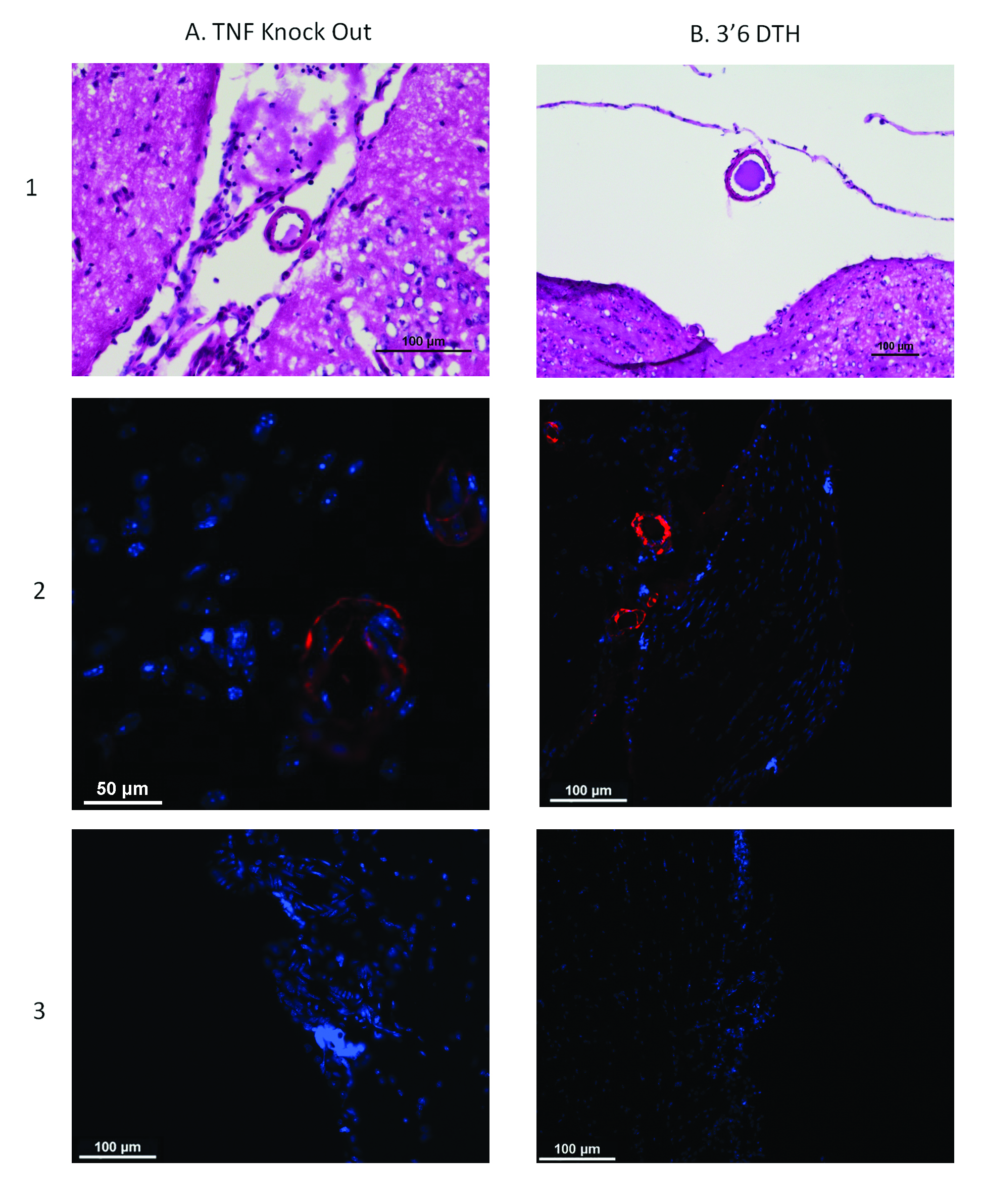
**

Representative hematoxylin and eosin images of TNF-α knockout mice (A1) and wild type mice treated with DTH (A2). Hematoxylin and eosin staining demonstrates normal cerebral vasculature with two to three layers of SMCs and a single, thin continuous layer of endothelial cells. Normal sized cerebral blood vessels stain only red for SM-22α in vascular segments (A2 and B2). TNF-α (green) expression is nearly non-existent (A2 and B2) and does not co-localize with SM-22α. Macrophages (red) are nearly non-existent (A3 and B3) and do not co-localize with TNF-α (green). Nuclei are stained blue with DAPI.

**Additional file1: Figure S2** **Beta-actin mRNA expression.  **

**
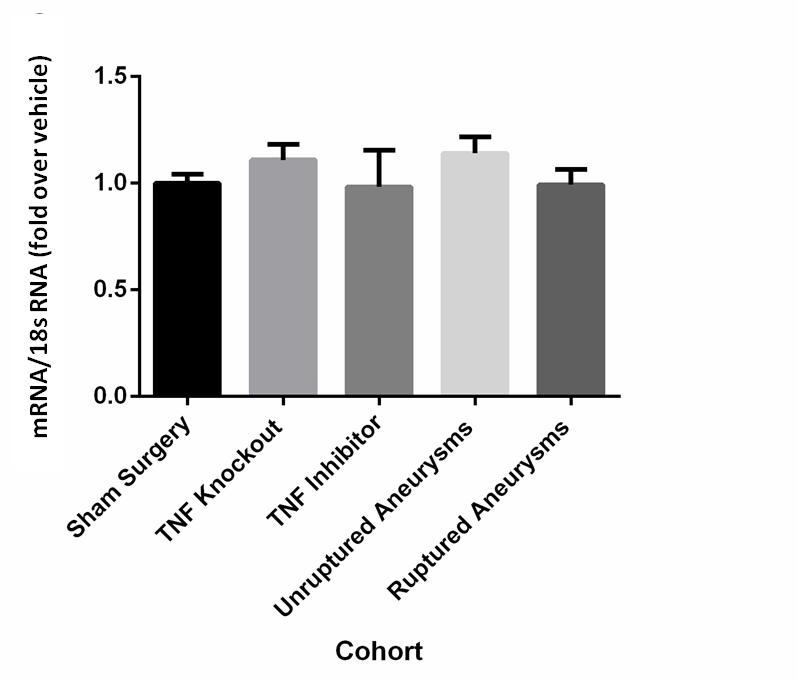
**

As compared with sham mice receiving vehicle, real-time PCR demonstrated no significant difference in beta-actin mRNA expression of cerebral blood vessels from TNF-α knockout mice, those pre-treated with DTH, unruptured aneurysms, or ruptured aneurysms.
